# Endoscopically placed fiducial markers for image-guided radiotherapy in preoperative gastric cancer: Technical feasibility and potential benefit

**DOI:** 10.1055/a-2129-2840

**Published:** 2023-09-21

**Authors:** Margot Bleeker, Astrid van der Horst, Arjan Bel, Jan-Jakob Sonke, Jeanin E. van Hooft, R. E. Pouw, Maarten C.C.M. Hulshof

**Affiliations:** 1 26066 Radiation Oncology, Amsterdam UMC, University of Amsterdam, Amsterdam, Netherlands; 2 Cancer treatment and quality of life, Imaging and biomarkers, Cancer Center Amsterdam, Amsterdam, Netherlands; 3 1228 Radiation Oncology, The Netherlands Cancer Institute, Amsterdam, Netherlands; 4 4501 Gastroenterology and Hepatology, Leiden University Medical Center, Leiden, Netherlands; 5 26066 Gastroenterology and Hepatology, Amsterdam UMC, University of Amsterdam, Amsterdam, Netherlands

**Keywords:** Diagnosis and imaging (inc chromoendoscopy, NBI, iSCAN, FICE, CLE), Endoscopy Upper GI Tract, Dilation, injection, stenting

## Abstract

**Background and study aims** Fiducial markers have demonstrated clinical value in radiotherapy in several organs, but little is known about markers in the stomach. Here, we assess the technical feasibility of endoscopic placement of markers in gastric cancer patients and their potential benefit for image-guided radiotherapy (IGRT).

**Patients and methods** In this prospective feasibility study, 14 gastric cancer patients underwent endoscopy-guided gold (all patients) and liquid (7 patients) marker placements distributed throughout the stomach. Technical feasibility, procedure duration, and potential complications were evaluated. Assessed benefit for IGRT comprised marker visibility on acquired imaging (3–4 computed tomography [CT] scans and 19–25 cone-beam CTs [CBCTs] per patient) and lack of migration. Marker visibility was compared per marker type and location (gastroesophageal junction (i.e., junction/cardia), corpus (corpus/antrum/fundus), and pylorus).

**Results** Of the 93 marker implantation attempts, 59 were successful, i.e., marker in stomach wall and present during entire 5-week radiotherapy course (2–6 successfully placed markers per patient), with no significant difference (Fisher’s exact test; *P* >0.05) in success rate between gold (39/66=59%) and liquid (20/27=74%). Average procedure duration was 24.4 min (range 16–38). No procedure-related complications were reported. All successfully placed markers were visible on all CTs, with 81% visible on ≥95% of CBCTs. Five markers were poorly visible (on <75% of CBCTs), possibly due to small marker volume and peristaltic motion since all five were liquid markers located in the corpus. No migration was observed.

**Conclusions** Endoscopic placement of fiducial markers in the stomach is technically feasible and safe. Being well visible and positionally stable, markers provide a potential benefit for IGRT.

## Introduction

Curative treatment of gastric cancer generally includes surgical resection combined with perioperative chemotherapy or postoperative chemoradiotherapy 1 2 . However, due to poor patient compliance during postoperative treatment regimens and uncertainties in target definition, research interest has recently shifted toward preoperative (chemo)radiotherapy 3 4 . For accurate image-guided radiotherapy (IGRT), localization of the target volume on pretreatment computed tomography (CT) imaging and daily in-room cone-beam CT (CBCT) imaging is essential. However, for the stomach, target localization can be challenging because of (organ) motion and low soft-tissue contrast. The use of fiducial markers potentially enhances target visualization during IGRT, which can aid target delineations 5 6 , and daily target localization and positioning 7 8 9 .

The feasibility and safety of fiducial marker placement have been demonstrated for various organs/cancers, including (upper) gastrointestinal sites 5 10 11 . Markers may improve IGRT accuracy, as demonstrated for pancreas 12 13 , rectum 14 15 , and esophagus 14 16 . For the stomach, however, marker implantation has only been investigated in a few small (case) studies 17 18 19 , with none covering visibility during IGRT courses. The stomach has distinct anatomical characteristics (i.e., hollow, deformable and experiencing peristaltic motion), which can affect implantation feasibility as well as marker visibility and stability on imaging. Therefore, more research regarding gastric fiducial markers is needed.

This prospective feasibility study aims to assess the technical feasibility and safety of endoscopic placement of (gold and liquid) fiducial markers in gastric cancer patients and their potential benefit during a 5-week IGRT course.

## Materials and methods

### Patient population

From October 2018 to January 2022, gastric cancer patients were enrolled in this prospective, non-randomized, single-arm feasibility study. Patient inclusion criteria were: histologically proven, stage IB-IIIC (TNM 8th edition), primary gastric adenocarcinoma, and referral for preoperative radiotherapy at our center. All eligible patients who were randomized to preoperative chemoradiotherapy within the CRITICS-II trial (NCT02931890) at our center were asked to participate in this fiducial study 3 . The ethics committee of the Amsterdam University Medical Center approved the protocol (study registration number NTR7241). Seventeen patients were eligible, 14 of whom gave written informed consent and were included. Included patients received fiducial marker placement in the stomach wall (not tumor) and additional imaging (CTs and CBCTs) during IGRT.

### Fiducial marker placement

All patients underwent endoscopy under conscious sedation with midazolam and fentanyl or under deep sedation with propofol. The procedures were performed by one of four experienced gastroenterologists. Intended marker locations were determined prior to implantation, at four to six stomach sites, taking distribution throughout the stomach and tumor location into consideration. Two different markers were used: the flexible 10-mm-long coil-shaped gold Visicoil marker (Visicoil, Core Oncology, California, United States; outer Ø=0.35 mm); and the liquid BioXmark marker (Nanovi A/S, Lyngby, Denmark). The liquid marker solidifies as a three-dimensional structure after implantation, thereby preventing diffusion. Gold markers were placed in all 14 patients; liquid markers were placed in the final seven patients once CE mark approval was obtained.

Gold markers were individually back-loaded into a 22G fine-needle aspiration needle, with the stylet pulled back about 2 cm; the needle tip was sealed with sterile bone wax to prevent accidental marker loss prior to implantation. For each gold marker, the loaded needle was placed in the gastroscope (GIF-HQ190; Olympus, Tokyo, Japan) for implantation. Following needle placement in the gastric wall, the marker was pushed out of the needle by pushing the stylet into the needle. For each marker, the needle was reloaded; sometimes, two needles were used to limit procedure time.

For the liquid marker, a 23G or 25G injection needle was primed prior to the injection procedure with 1 mL of liquid marker followed by saline solution until the injection system was fully filled. Next, multiple consecutive markers were placed into the gastric wall, using a unit dose syringe (Luer Lock, Vlow medical, Eindhoven, The Netherlands) for controlled dosage of injected volume. For fiducial markers in the esophagus, a volume of >0.05mL proved sufficiently visible on CT and CBCT 16 ; however, as the stomach possibly experiences more movement during imaging, we aimed for approximately 0.1mL of injected volume per marker. The needle was maintained in the tissue for about 5 seconds because of slow release of the viscous liquid marker. Fluoroscopy was sometimes used to check marker placement.

### Image-guided radiotherapy

The target for IGRT was the entire stomach and regional lymph nodes. IGRT (45 Gy in 25 fractions) treatment planning was based on a reference CT scan. For all patients except one, this reference scan was acquired after implantation (0–5 days, median 1 day). During the IGRT course, daily CBCTs and three repeat CTs were acquired. Median time between implantation and the first and last CBCT scan was 13 days (range 5–28) and 46 days (range 37–60), respectively. For details on chemoradiotherapy, see **Supplemental Material 1** .

### Outcome measures

#### Technical feasibility

Following each marker implantation, the gastroenterologist assessed expected success of placement. A marker was successfully placed when placed in the stomach wall and present for the entire course of IGRT (i.e., at time of the reference scan, and the first and last CBCT scan). Successful placement was assessed for all markers, per marker type and for three sites: gastroesophageal junction (i.e., gastroesophageal junction/cardia), corpus (i.e., corpus/antrum/fundus), and pylorus. Also, the technical difficulty of implantation (i.e., easy, reasonable, or difficult) was scored. Finally, the duration of the entire procedure (from first loaded needle entering the gastroscope to final marker placed) and average time per attempted marker implantation were assessed.

### Adverse events

For each marker, the gastroenterologist scored whether bleeding occurred at the implantation location. Potential adverse events (AEs) occurring in the first 24 to 48 hours following implantation were registered. The following AEs were potentially procedure-related: bleeding, infection/fever, and pain.

### Potential benefit

The potential benefit of markers for target delineation and position verification during IGRT depends upon their visibility on (CB)CT scans and positional stability throughout radiotherapy. For each successfully placed marker, visibility was separately assessed for each available CT and CBCT scan. Marker visibility was defined as good (marker visible on ≥95% of scans), moderate (on ≥75%), or poor (<75%). Marker visibility was assessed for the three sites: gastroesophageal junction, corpus, and pylorus. For analyses of marker visibility on respiratory phase images, see **Supplemental Material 2** . Positional stability was defined as lack of observable migration within the tissue.

Technical feasibility and potential benefit were compared between gold and liquid markers.

### Statistical analyses

Feasibility of marker implantation was assessed using descriptive statistics. Ratios were compared with the two-sided Fisher’s exact test (α=0.05).

## Results

### Technical feasibility

In the 14 patients, 93 endoscopy-guided markers implantations attempts were performed (66 gold and 27 liquid markers; Table 1 ). For each liquid marker, a volume of 0.08 to 0.20mL was injected.

**Table:** **Table 1** Patient, tumor and procedure characteristics, combined with the number of successful implantations.

No.	Age (years)	Sex	Tumor location	No of attempts, type of markers	Successful implantation	Procedure duration (minutes)	Fluoroscopy used
1	62	M	Cardia	7 gold	4 gold	38	Yes
2	38	M	Antrum	5 gold	3 gold	25	Yes
3	48	M	Corpus and pylorus	6 gold	3 gold	16	Yes
4	70	M	Corpus and antrum	5 gold	4 gold	19	Yes
5	65	M	Antrum	7 gold	6 gold	19	Yes
6	71	M	Antrum and pylorus	6 gold	3 gold	21	No
7	58	F	Corpus	6 gold	4 gold	24	No
8	48	M	Cardia	4 gold; 4 liquid	1 gold; 3 liquid	27	No
9	45	M	Cardia	3 gold; 3 liquid	2 gold; 2 liquid	30	No
10	56	F	Antrum	4 gold; 4 liquid	2 gold; 4 liquid	20	No
11	64	M	Cardia and distal esophagus	5 gold; 3 liquid	2 gold; 3 liquid	24	No
12	69	M	Corpus	3 gold; 4 liquid	3 gold; 3 liquid	24	Yes
13	61	M	Cardia	3 gold; 4 liquid	2 liquid	33	No
14	60	M	Antrum and pylorus	2 gold; 5 liquid	2 gold; 3 liquid	21	No
Total	Median (range) 61 (38–71)			66 gold; 27 liquid	39 gold; 20 liquid	Mean (range) 24.4 (16−38)	

**Fig. 1:**
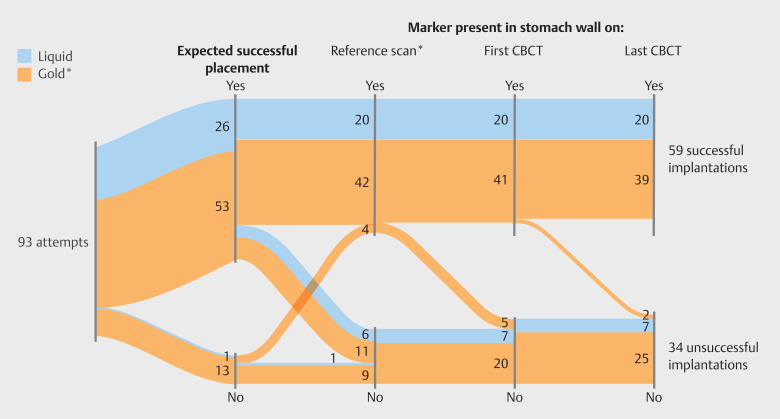
Sankey plot showing the number of implantation attempts (N=93), the number of marker implantations expected to be successful (N=79), the number of markers placed in the stomach wall and present on the reference scan (N=66), the first CBCT (N=61), and the last CBCT (N=59). Seventeen markers expected to be successfully placed were either not visible on the reference scan (N=10) or not placed in the stomach wall (N=7). *For one patient, the reference scan was acquired prior to marker implantation; for this patient, we regarded the first CBCT also as reference scan.

Fifty-nine markers (63%; 2 to 6 per patient) were successfully placed ( Fig. 1 ); this was 59% and 74% for gold and liquid markers, respectively. No significant difference was found between success rates for gold and liquid markers ( *P* >0.05) or when only comparing the seven patients with both marker types. Moreover, success rates differed between sites as it was 63% (10 successfully placed of 16 attempts) for markers in the gastroesophageal junction, 60% (37/62) in the corpus, and 80% (12/15) in the pylorus. Of the unsuccessful implantations, 17 markers that were expected to be successfully placed were either not visible on the reference scan (N=10; lost in the first 0 to 5 days following implantation) or not placed in the stomach wall (N=7; 5 gold and 2 liquid markers); of these latter seven, three were placed outside the stomach wall (<1cm), one in the spleen, one in the diaphragm, and two in surrounding fat. All seven markers placed outside the stomach wall were present for the entire radiotherapy course. In addition, five markers were lost between the reference scan (on days 0–3 following implantation) and the start of IGRT delivery (i.e., first CBCT; on days 9–14 following implantation), and two were lost during IGRT delivery (on days 15 and 22 post-implantation). Of the 93 attempts, 14 were not expected to be successfully placed during implantation (13 gold and 1 liquid) because, for instance, the marker was partially sticking out of the mucosa into the lumen, the marker could not be pushed out of the needle, or liquid marker that leaked intraluminally from the tissue. Of the 14 markers expected not to be successfully placed, four were visible on the reference scan; three of these four were lost prior to the end of IGRT.

The average procedure duration was 24.4 minutes (range 16–38 minutes), with time per marker attempt 2.5 to 5.4 minutes (average 3.7 minutes). The technical feasibility was rated easy for 77 markers, reasonable for nine, and difficult for seven. All difficult implantations were unsuccessful. Reasons for difficult marker implantation included challenges with pushing the gold or liquid marker out of the needle; causes included sharp angulation of the gastroscope or changes in bone wax type/brand.

### Adverse events

Only mild bleeding occurred (N=15, all gold), indicating that the bleeding stopped immediately. No procedure-related complications were reported afterwards.

### Potential benefit

**Fig. 2:**
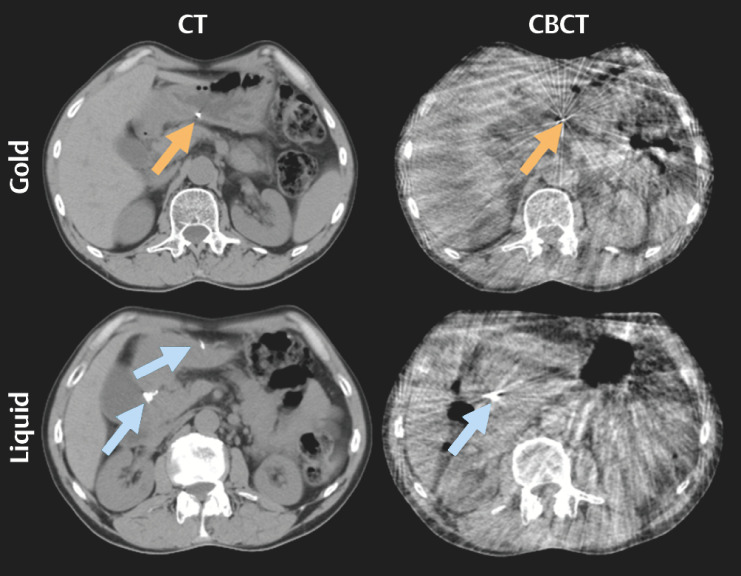
Typical example of gold (orange arrows) and liquid (blue arrows) fiducial markers on CT and CBCT. On the CTs, two liquid markers are visible; one of the two was, although present, not visible on the CBCTs.

**Fig. 3:**
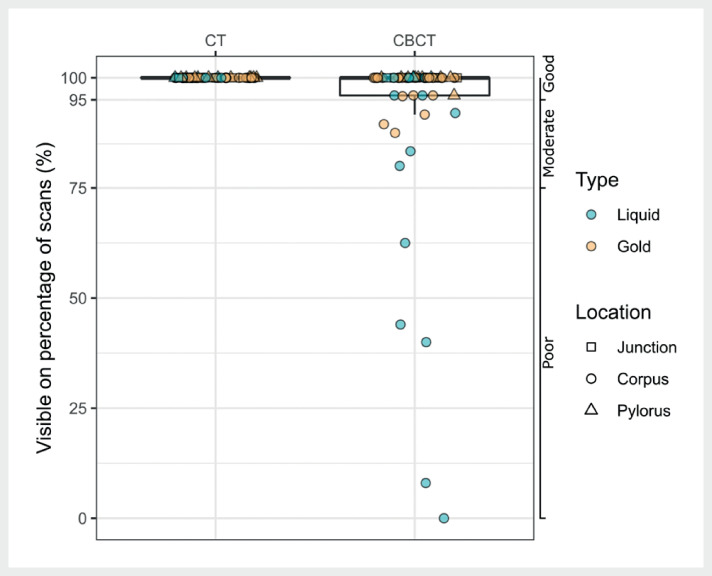
Boxplots of the visibility of successfully placed markers (N=59) on CT and CBCT scans for liquid (blue) and gold markers (orange). Symbols indicate marker location: square = gastroesophageal junction (i.e., junction), circle = corpus, triangle = pylorus). Boxplots: box = interquartile range (IQR), whiskers = lowest and highest data point within 1.5×IQR.

For each patient, three to four CTs (total=53) and 19 to 25 CBCTs (total=339) were used to score marker visibility ( Fig. 2 ). The visibility of all successfully placed markers was good on CT scans ( Fig. 3 ). On CBCTs, most markers (81%) had good visibility. For only five markers (four patients), visibility was poor on CBCT. All five were liquid markers (significantly more often than gold marker; *P* =0.003) located in the corpus (not significant compared to the other locations); three were visually assessed as small.

Besides marker loss between the first and last acquired scan, no apparent migration within the tissue was observed.

## Discussion

Currently, there is limited research on fiducial marker implantations in the stomach. Hence, in this prospective feasibility study, we demonstrated that endoscopic fiducial marker placement in the stomach is technically feasible and safe. In addition, the successfully implanted fiducial markers are positionally stable and sufficiently visible on acquired imaging, thereby showing their potential benefit.

Implantation was successful in 63% of implantation attempts, with no significant difference in success rates between gold and liquid markers. Every patient had at least two markers (range 2 to 6) successfully implanted in the stomach wall that remained present for the entire IGRT course (i.e., 37 to 60 days post-implantation). In similar studies for gastrointestinal cancers 11 12 13 17 20 21 , technical success is often measured by the ability to successfully place at least two markers in the target. With this measure, our technical success rate per patient is 100%, which is similar to or higher than previous studies that include gastric cancer 17 21 . The characteristics of the stomach (e.g., hollow and flexible with peristaltic motion) may complicate implantations, as reflected in the success rate of 60% in the corpus.

Similarly, implantations in the gastroesophageal junction were occasionally challenging due to the retroflexed position of the gastroscope (success rate 63%). In contrast, implantations in the pylorus, with a success rate of 80%, were generally more straightforward. However, although some implantations were unsuccessful, these were regularly expected to be unsuccessful during the procedure (N=14; e.g., gold markers partially sticking out of the tissue), and additional markers could still be placed at the same location. Hence, the clinical impact of these unsuccessful implantations on IGRT was minimal.

During this study, the implantation procedures were found to be generally efficacious. A large number of implantation attempts (5–8) and successful implantations (2–6) per patient were performed within a relatively short implantation procedure duration (24.4 minutes). Furthermore, few technical difficulties were encountered and four different gastroenterologists performed the implantations, thereby showing broad applicability. In addition, similarly to other studies in which these gold (Visicoil) and/or liquid markers (BioXmark) were implanted, we found no procedure-related complications 11 16 21 . Hence, we demonstrated that fiducial marker implantations in the stomach are technically feasible and safe.

Most successfully placed markers had good visibility on both CT and CBCT imaging. Poor visibility on CBCT was likely primarily caused by marker location (i.e., corpus has largest peristaltic motion) and/or type (i.e., small-volume liquid marker). Migration within the tissue was not observed but is also difficult to assess because of the large day-to-day stomach shape changes. In this feasibility study, markers were placed distributed throughout the stomach and frequently also near the tumor borders. Even though in this study the target for IGRT included the entire stomach, these markers can evidently also assist in tumor demarcation when the tumor is the intended target. Hence, with good overall marker visibility and no marker migration, fiducial markers in the stomach show great potential to improve target delineations, and daily target localization and positioning during gastric cancer IGRT. Consequently, by implanting fiducial markers, radiotherapy accuracy may be enhanced, thereby potentially contributing to reduced toxicity and improved treatment outcomes for patients with gastric cancer.

Including patients with both marker types, although only seven, enabled fair comparisons of the implantation procedure, the number of successful implantations, and marker visibility, unlike other studies with multiple marker types 13 20 21 . For liquid markers, multiple markers could be placed without retraction and reloading of the needle. Therefore, in addition to being user-friendly, liquid markers have the potential to reduce procedure time and associated costs. Moreover, when multiple markers are required, the cost of liquid marker implantations may be further reduced compared to gold markers as multiple markers can be implanted from a single purchased 1-mL ampoule. Conversely, despite the use of a unit dose syringe to regulate the injected liquid marker volume, various marker volumes were observed (e.g., very large and too small volumes). By comparison, gold markers always contain the same amount of gold and have a higher contrast due to the higher density. Gold markers are thus more consistently visible in imaging and easier to locate. However, the visibility of liquid markers may be enhanced by clinical implementation of advanced CBCT reconstruction methods 22 or by ensuring that an adequate volume of the liquid marker is injected 16 . Hence, each marker has its benefits, and the type of marker should be selected based on the intended use.

Strengths of this study include the clear focus on the stomach, the relatively large number of implanted fiducial markers, and the extensive evaluation of marker visibility on imaging data. As a result, we were able to show the evident clinical applicability of fiducial markers during IGRT, despite the relatively small patient cohort in this feasibility study. Because liquid markers were placed only in the final seven patients due to the absence of CE mark approval at the start of patient inclusion, this study contained unequal sample sizes of gold and liquid markers. Moreover, because each patient had multiple markers, the statistical tests included non-independent observations. Hence, statistical comparisons between marker types and locations should be cautiously interpreted. Furthermore, marker implantations were performed in this study without endoscopic ultrasound (EUS) guidance, unlike similar studies. Limiting factors of EUS guidance can be its technical characteristics (e.g., less flexible scope) and lack of widespread availability 17 . Although implantations outside the stomach wall occurred for only a small number of markers (N=7), EUS guidance may prove beneficial in preventing such unsuccessful implantations. A greater positive impact on the success rate in general may be expected from more clinical experience by the gastroenterologist in both needle loading/sealing and marker injection or consistent use of fluoroscopy.

## Conclusions

In conclusion, fiducial marker implantations, both gold (Visicoil) and liquid (BioXmark), were feasible and safe in gastric cancer patients. Furthermore, because they have good overall visibility on the acquired images during 5-week IGRT, gastric fiducial markers have potential benefit for radiotherapy.

## Supporting information

Supplementary material Zusatzmaterial
