# Supplementary material for: Endoscopically placed fiducial markers for image-guided radiotherapy in preoperative gastric cancer: Technical feasibility and potential benefit
Source: Endosc Int Open. 2023 Sep 21;11(9):E866–72. doi: 10.1055/a-2129-2840 (PMC10513787; doi:10.1055/a-2129-2840)

Supplementary material

Supplemental material 1: Chemoradiotherapy course

For all included patients, chemoradiotherapy (CRT) consisted of 45 Gy in 25 fractions, combined with weekly paclitaxel and carboplatin. Image-guided radiotherapy (IGRT) treatment planning was based on a reference computed tomography (CT) scan (resolution 0.98×0.98×2.5 mm^3). For all patients except one, the reference scan was acquired after implantation (0−5 days, median 1 day); for the one patient, the reference scan was acquired two days before implantation. During IGRT delivery, daily cone-beam CTs (CBCT; 1.00×1.00×1.00 or 0.91×0.91×1.00 mm^3) were acquired in the treatment position at the linear accelerator for position verification, as is typically done in our institute. Such CBCTs are low-quality 3D images that enable positioning on bony anatomy and, when visible, verification of target coverage using the fiducial markers. The CBCT acquisition time was approximately 4 minutes, thereby including multiple respiratory cycles and potential peristalsis. Furthermore, within this feasibility study, repeat CTs were acquired in the first, third and fifth week of radiotherapy.

For patients 1−5, CTs were acquired with the GE LightSpeed RT16 CT and CBCTs were acquired with the Elekta Infinity linear accelerator. For patients 6−14, CTs were acquired with the GE Discovery CT590 RT CT and CBCTs with the Varian TrueBeam linear accelerator.

Supplemental material 2: Marker visibility on respiratory phase scans

4DCTs and 4DCBCTs consist of a series of scans that each represent a different respiratory phase. Fiducial markers that are visible on respiratory phase scans can enable target respiratory motion assessments, which may aid in the accurate delivery of IGRT. Thus, marker visibility was also evaluated for such respiratory phase scans.

For CT and CBCT, 10 respiratory phase scans were reconstructed. As the same data is used to reconstruct the average scan and the 10 respiratory phase scans, image quality of the individual respiratory phase scans is lower than that of the average scans. For each marker, marker visibility was assessed on each end-inhale and end-exhale scan (Supplemental Figure 1). For respiratory phase scans, a marker was only regarded visible when it was visible on both end-exhale and end-inhale scans.

For CT scans, whereas all markers had good visibility on average scans, 5 gold markers were moderately visible on respiratory phase scans. Furthermore, for CBCT scans, there were 5 and 10 poorly visible markers on average and phase scans, respectively.

Supplementary material

Supplemental figure 1: Boxplots of the visibility of successfully placed markers (N=59) on CT and CBCT average and phase scans, for liquid (blue) and gold markers (orange). A marker was regarded visible on a phase scan, when present on both end-inhale and end-exhale scans. Symbols indicate marker location: square = gastroesophageal junction (i.e., junction), circle = corpus, triangle = pylorus). Boxplots: box=interquartile range (IQR), whiskers=lowest and highest data point within 1.5×IQR.

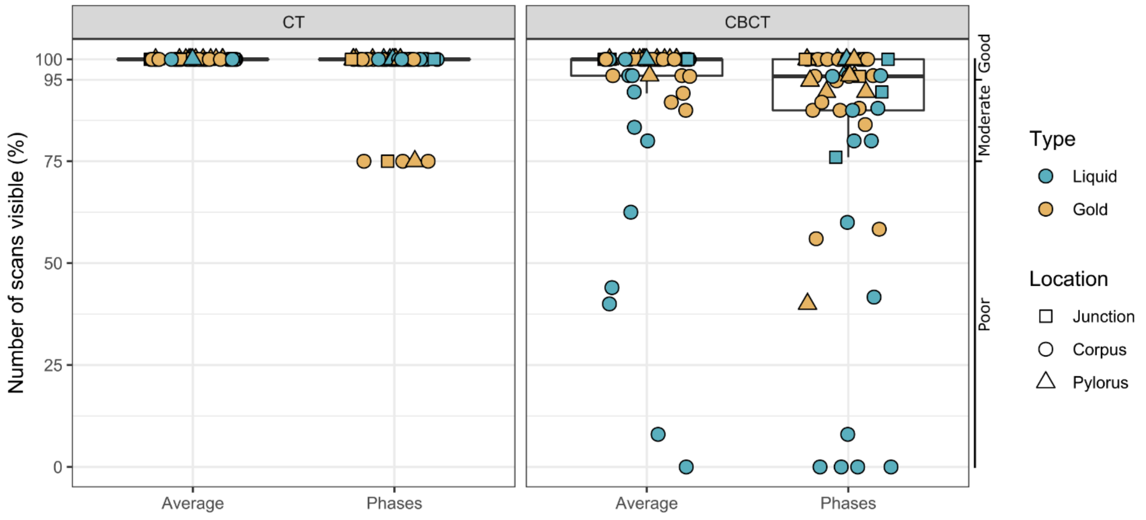

Supplement: Supplementary file 1 — Supplementary material [file 10-1055-a-2129-2840_21454811.pdf]
